# Diffusion-weighted imaging of the brains of dogs with idiopathic epilepsy

**DOI:** 10.1186/s12917-017-1268-0

**Published:** 2017-11-15

**Authors:** Antje Hartmann, Steffen Sager, Klaus Failing, Marion Sparenberg, Martin J. Schmidt

**Affiliations:** 1 Department of Clinical Veterinary Medicine, Vetsuisse Faculty, University of Bern, Länggassstraße 128, 3012 Bern, Switzerland; 2 Department of Veterinary Clinical Sciences, Small Animal Clinic, Justus-Liebig-University Giessen, Frankfurter Straße 108, 35392 Giessen, Germany; 3 Unit for Biomathematics and Data Processing Faculty of Veterinary Medicine Justus Liebig-University, Frankfurter Straße 95, 35392 Giessen, Germany

**Keywords:** Magnetic resonance imaging, Canine, MRI, Apparent diffusion coefficient, ADC, Seizure

## Abstract

**Background:**

Idiopathic epilepsy is one of the most common neurological disorders in dogs. Unfortunately, up to 30% of dogs with idiopathic epilepsy show no improvement under antiepileptic drug treatment. Diffusion-weighted imaging is used in human medicine to identify epileptogenic foci in the brain to allow for more invasive treatments such as deep brain stimulation or surgical removal.

The aim of this study was to ass the feasibility of interictal diffusion-weighted MRI in dogs and to evaluate the distribution of diffusion in the brains of dogs with idiopathic epilepsy (IE) and to compare these values to previously published values from healthy beagle dogs.

Client-owned dogs with the final diagnosis of IE were included in this study. MRI examination was carried out using a 1.0Tesla superconductive magnet. Diffusion-weighted images using a single shot echo planar imaging sequence (SSh-EPI) with a b value of b = 0 s/mm^2^ and b = 800 s/mm^2^ were acquired in a dorsal and transverse plane with diffusion gradients in all three planes (x-, y- and z-plane). An ADC (apparent diffusion coefficient) map of the isometric image of each acquired slice was generated.

Regions of interest (ROIs) were manually drawn around the caudate nucleus, the thalamus, the piriform lobe including the amygdala, the hippocampus, the semioval center and the temporal cerebral cortex by one of the authors. ROI drawings were repeated 5 times at different time points to assess intra-obersver variability. A multi-way mixed-model analysis of variance (ANOVA) and two-way ANOVA were used during statistical analysis. A *p* value of *p* < 0.05 was considered significant.

**Results:**

Dogs with IE showed a significantly increased ADC in the amygdala within the piriform lobe and in the semioval center (p < 0.05) compared with the healthy control group.

**Conclusion:**

Changes in the piriform lobe in cases of epilepsy are reported infrequently in human and veterinary medicine. Similar to our results, ADC changes in the interictal phase usually include an increase in ADC due to cell loss and increased intercellular spaces. Diffusion MRI might be a promising technique for the examination of canine epileptic patients lacking other gross neuromorphological abnormalities.

## Background

Idiopathic epilepsy (IE) is considered the most common neurological disorder in dogs, with a reported incidence of 0.5–5% [1–4]. IE is characterized by the presence of recurrent seizures without any underlying clinical disorder causing the seizures; thus, a genetic basis is presumed [1, 5, 6]. Dogs usually exhibit a normal neurological examination in the interictal phase. The diagnosis of IE is based on the exclusion of other diseases [1, 7–9]. The onset of the disease in dogs is usually between 1 and 6 years, but an onset between 6 months and 10 years is possible [1, 8, 9]. Lifelong medication with antiepileptic drugs is necessary, however, treatment failure can occur in up to 30% of dogs [1, 10]. These unresponsive dogs become an increasingly critical issue, and innovative diagnostic and therapeutic strategies will be needed in the future.

Analogous to human patients [11], the concept of an epileptogenic zone has been proposed for dogs as a functional area of the cortex initiating and propagating seizures that cannot be defined by anatomical imaging [12]. Surgical resection or isolation of the brain area that triggers the seizures, e.g.*,* partial or total temporal lobe resection or subpial transection, represent more invasive treatment options in human patients with refractory epilepsy. Such procedures can lead to seizure reduction [13–20]. For temporal lobe epilepsy (TLE) in particular, early surgery for patients with mesial TLE is superior to medical therapy [21]. Epilepsy surgery has also begun to attract veterinary attention [22]. However, surgical techniques require presurgical mapping of the cortex and the exact localization of the epileptogenic zone. In dogs with no gross neuromorphologic abnormality diffusion-weighted imaging might be employed to help visualize patho-physiological processes in the brain parenchyma [23, 24]. Diffusion-weighted imaging (DWI) has been used to detect the epileptogenic zone in human epileptic patients and in animal models of epilepsy [12, 25, 26].

DWI measures the degree of thermal motion of a substance based on Brownian motion within the imaged tissue. In the brain, the movement of water molecules in three compartments contributes to the measured degree of diffusion: intravascular perfusion, extracellular diffusion and intracellular diffusion. Thus, the measured degree of diffusion not only includes diffusion-based motion but also perfusion. In addition, a variety of factors influence the motion of water molecules in tissues. To acknowledge this variation, the term apparent diffusion coefficient (ADC) is used [27–29].

Reduction in diffusion in human patients with temporal lobe epilepsy is described during the ictus and postictally [30], whereas increased diffusion has been described in cases of presumed hippocampal sclerosis [31]. An experimental study of dogs showed an initial decrease (3–6 h) in diffusion after kainic acid-induced complex partial status epilepticus, which was followed by an increase in diffusion after 12 and 24 h and a normalization after 48 h. The authors suggested that diffusion-weighted imaging can be useful to detect the epileptogenic focus or assess potential epileptic brain damage in dogs [25].

The aim of our study was to assess the feasibility of interictal DWI to detect areas with altered diffusion in comparison to healthy control dogs and to analyze the distribution of diffusion in the brain of dogs with idiopathic epilepsy (IE).

## Methods

### Study design

The study was conducted prospectively. All investigations were performed in strict compliance with the restrictions of the German Animal Protection Law. All dogs were client-owned and lived with their owners. The owners of the dogs gave permission for their animals to be used in this study. Approval from the Committee on the Ethics of Animal Experiments of the Justus-Liebig-University and the local Hessian government was not required as all examinations were performed for diagnostic reasons at the owner’s request. All owners were informed that the data of their dogs might be used for this study, which they agreed upon. The MRI protocol was similar to a previous study that was approved by the Committee on the Ethics of Animal Experiments of the Justus-Liebig-University Giessen and local Hessian government (reference number: V54-19c2015(1)GI18/17 Nr. 78/2011) [32].

### Study population and MRI examination

During the study period (January 2013 to January 2014), 89 dogs were presented to the neurology unit of the Justus-Liebig-University of Giessen because of seizures. Only dogs with a final diagnosis of IE were included in this study. IE was defined following the proposal of the international veterinary epilepsy task force [9] as recurrent, generalized, tonic-clonic seizures with autonomic clinical signs (salivation, urination) and an impaired state of consciousness during the previous 4 weeks. Dogs showing a single seizure, partial seizures or complex partial seizures were not included. The classification of seizures was performed by video analysis.

Dogs must have been between 1 and 3 years old when the first seizures occurred. The general and neurological examination carried out by a board certified neurologist needed to be normal interictally. Laboratory results (complete blood count, serum chemistry profile and serum ammonia, pre- and postprandial serum bile acids), standard MRI and the CSF analysis needed to be unremarkable. Dogs with a history of dystocia during birth, head trauma and dogs that received antiepileptic drug treatment were excluded from the study.

The anesthetic protocol and MRI system were identical to the settings published for normal dogs using a using a 1.0 Tesla^1^ superconductive system and a SENSitivity Encoding (SENSE) coil^2^ [32]. The following sequences were acquired: dorsal, sagittal and transverse T2-weighted images (TE: 85 ms; TR: 4000 ms), transverse T2-weighted FLAIR images (TE: 97.5 ms; TR: 3962 ms; TI: 2000 ms), transverse T2*-weighted gradient echo images (TE: 21 ms; TR: 454 ms), transverse T1-weighted images (TE: 15 ms TR: 593 ms) and dorsal 3D T1-weighted gradient echo images (TE: 6.9 ms; TR: 25 ms). T1-weighted images were acquired pre- and post-contrast medium administration. Diffusion-weighted images using a single shot echo planar imaging sequence (SSh-EPI) with a b value of b = 0 s/mm^2^ and b = 800 s/mm^2^ were acquired in a dorsal and transverse plane with diffusion gradients in all three planes (x-, y- and z-plane) before contrast medium administration identical to the published protocol in normal dogs [32]. The slice orientation was parallel to the base of the skull for dorsal images and perpendicular to it for transverse images. Slices were oriented such that one slice crossed the thickest part of the caudate nucleus.

Further image processing was identical to the published processing in normal dogs. Isometric images including the diffusion effects from all three image planes were automatically generated. Using the integrated software, an ADC (apparent diffusion coefficient) map of each acquired slice was generated [32].

**Fig. 1:**
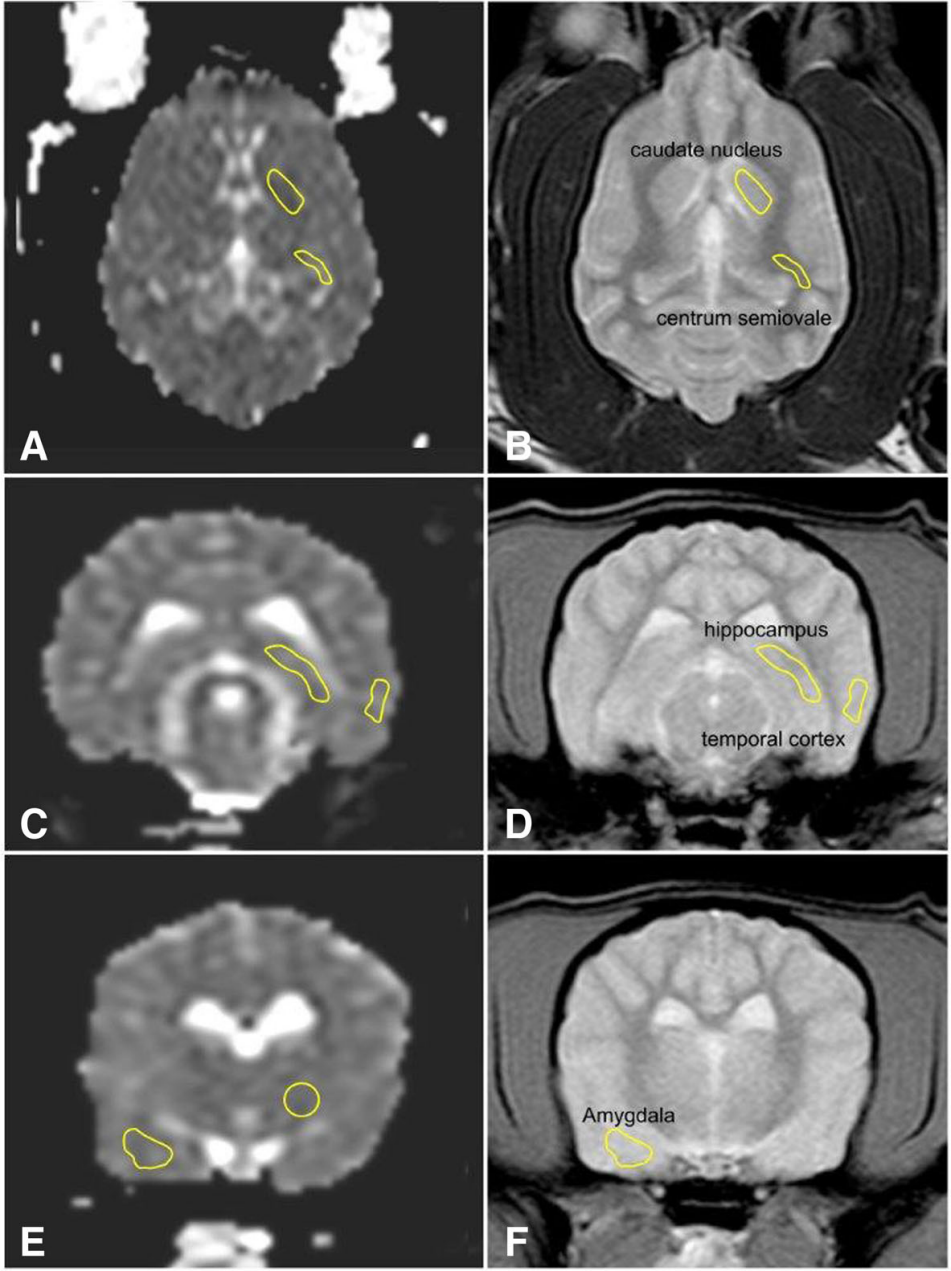
Diffusion weighted images (**a**, **c**, **d**) and corresponding T2-weighted images (**b**, **d**, **f**) showing the ROI placement around the caudate nucleus and semioval center (**a** + **b**), the hippocampus and the temporal cerebral cortex (**c** + **d**), the amygdala in the piriform lobe and the thalamus (**e** + **f**)

A drawing of the region of interest (ROI) was performed in accordance with a previously published study in healthy dogs. ROIs were manually drawn around the caudate nucleus, the thalamus, the piriform lobe including the amygdala, the hippocampus, the semioval center and the temporal cerebral cortex [32] (Fig. 1). Subjective visibility of the structure on the respective image determined whether a transverse or a dorsal image was used to draw the region of interest (ROI). Most structures were only visible on one slice. If a region was visible on more than one slice, the slice that showed the largest extent of the region was used. Images of the other sequences were available as anatomical references at time of ROI placement. The ROIs were drawn as large as possible while avoiding the inclusion of other structures. The ROI drawings were repeated 5 times at different time points. The integrated software calculated the ADC in μm^2^/s (= 10^−6^ mm^2^/s) [32]. All ROI drawings were performed by the second author of this study. Time gap between the individual measurements was 3–4 weeks.

Statistical analysis was performed using the software BMDP/Dynamic, Release 8.1 [32, 33]. A two-way analysis of variance (ANOVA) with repeated measures was used to evaluate the effect in diseased versus healthy dogs by comparing the results of this study to values obtained earlier in healthy beagle dogs (program BMDP2V; ANOVA and covariance with repeated measures). The results from the previous study in healthy beagle dogs are already published [32]. The level of significance was *p* < 0.05 for all tests.

For consistency with our first study about brain diffusion in healthy dogs and for being able to better compare possible differences intra-observer variability, the effect of the brain side (right versus left), as well as assessing differences between the different anatomic regions were assessed in addition. The intra-observer variability was evaluated by calculating the mean variation by a multi-way mixed-model analysis of variance with a random observer effect (program BMDP8V). The related coefficient of variation [%] was calculated by dividing this standard deviation by the grand mean multiplied by 100. A two-way analysis of variance (ANOVA) with repeated measures was used to examine the effect of anatomical region and side (left versus right) (program BMDP2V; ANOVA and covariance with repeated measures). A multi-way mixed model analysis of variance (program BMDP8V) was used to determine the region-specific standard deviation with respect to repeated measurements and to perform a region-specific side (left versus right) comparison.

## Results

Seventeen dogs met the inclusion criteria. The mean age of the dogs was 3.09 ± 2.35 years. The mean body weight 29.23 ± 8.28 kg (Table 1).

**Table 1:** Characteristics of included dogs

Breed	Age in years	Weight in kilograms	Sex	Time between last seizure and MRI in days
Australian Shepherd	8	28	mn	1
Giant Schnauzer	2	35	m	1
Airedale terrier	3	29	mn	1
Border Collie	1	21	m	1
Landseer	2	47.1	w	8
Mixed breed	1	14.2	w	2
Labrador retriever	9	35	mn	1
Entlebucher Mountain Dog	4	38.5	m	2
French bulldog	2	15	mn	2
Labrador retriever	2	26.5	m	4
Mixed breed	1	31.9	w	2
Wirehaired Pointer	4	25	w	9
Mixed breed	2	33	mn	1
Border collie	4	21.7	m	1
Golden retriever	3	32	m	2
Labrador retriever	1	33	w	2
Golden retriever	4	31	m	3

The caudate nucleus had an ADC of 871.47 ± 179.67 μm^2^/s (mean ± standard deviation (SD)). The ADC of the thalamus was 811.51 ± 175.96 μm^2^/s. The piriform lobe including the amygdala had an ADC of 963.80 ± 174.69 μm^2^/s. The hippocampus had an ADC of 1015.03 ± 231.79 μm^2^/s. The ADC of the semioval center was 790.98 ± 229.63 μm^2^/s. The temporal cerebral cortex had an ADC of 875.65 ± 225.48 μm^2^/s.

Significant differences were found comparing the data from this study to previously published data in normal dogs for the piriform lobe including the amygdala (*p* = 0.02) and the semioval center (*p* < 0.01). Diffusion was increased in the piriform lobe and the semioval centre in diseased dogs compared to normal dogs.

The caudate nucleus and the piriform lobe showed significant differences between the right and left side. With higher values for the left sided structures. However, not all regions showed higher values in the left side. Table 2 gives an overview of the ADC values, including minima, maxima and range for the right and left cerebral hemisphere individually.

**Table 2:** ADC values given in μm^2^/s of the different brain regions listed by side for diseased and healthy dogs

Region	Seite	Mean ADC [μm^2^/s]		SD ADC [μm^2^/s]		Min ADC [μm^2^/s]		Max ADC [μm^2^/s]	
		diseased	normal	diseased	normal	diseased	normal	diseased	normal
Caudate nucleus	Right	836.44^a^	843.3	172.26	94.3	761.08	751.7	982.58	1089.5
	Left	906.5^a^	962.4	187.08	103.2	827.13	864.4	1140.69	1125.0
Thalamus	Right	819.8	792.2	167.14	31.0	767.29	741.7	893.92	833.2
	Left	803.22	823.4	184.78	30.1	706.33	794.9	868.62	874.8
Piriform lobe^c^ (incl. Amygdala)	Right	958.96^b^	895.0	184.23	50.6	856.72	806.0	1086.76	962.5
	Left	968.64^b^	935.9	165.15	37.7	869.62	898.0	1049.03	1008.3
Hippocampus	Right	1027.93	1052.9	230.04	109.7	882.39	902.4	1230.62	1236.3
	Left	1002.14	1035.7	233.54	109.0	880.96	919.5	1138.59	1270.8
Semioval center∆	Right	786.36	717.6	241.4	73.8	641.39	597.0	894.95	808.9
	Left	795.59	725.2	217.85	83.0	666.33	609.5	893.94	851.8
Cerebral cortex	Right	866.16	820.2	236.35	71.5	763.57	738.5	975.18	960.6
	Left	885.14	857.1	214.61	63.4	761.81	809.0	1055.38	962.6

Significant differences between the right and left cerebral hemisphere were found for the caudate nucleus (*p* < 0.0001) and the piriform lobe including the amygdala (*p* < 0.01). Significant differences between normal and diseased dogs were found for the piriform lobe including the amygdala and for the seminoval centre. Normal values are from the publication about normal dogs [32]

*ADC* apparent diffusion coefficient, *Max* maximum ADC, *Min* minimum ADC, *SD* standard deviation; ^a^ highly significant difference between right and left hemisphere <0.0001; ^b^significant difference between right and left hemisphere *p* < 0.01; ^c^significant difference between normal and diseased dogs *p* = 0.02; ∆ significant difference between normal and diseased dogs *p* < 0.01

The intra-observer variability was slightly higher as for normal dogs (intra-observer variability in normal dogs in general 2% [32]). With an average variation of 2.8% with the greatest degree of variation occurring in the semioval center and the least degree of variation in the piriform lobe.

## Discussion

Diffusion changes in epilepsy are well documented in human medicine [34–36] and have also been described in an experimental study of kainic acid-induced complex partial status epilepticus in dogs [25]. In general, a transient reduction in diffusion with low ADC values is described. Changes are considered to be the result of cytotoxic or intramyelinic edema [25, 30, 31, 36]. A significant difference was found in the diffusion between normal and idiopathic epileptic dogs in the piriform lobe including the amygdala and the semioval center, with significantly higher ADC values in affected dogs.

The diagnosis of idiopathic epilepsy was made by the exclusion of other diseases based on a normal general and neurological examination and normal blood work, as well as by standard MRI and cerebrospinal fluid analysis. Dogs with a history of dystocia during birth, or head trauma or dogs that had previously received AED where excluded to avoid any effect on our results. The dogs examined were between 1 year and 9 years old. The usual age at which dogs present with idiopathic epilepsy ranges from 1 to 6 years; however, an onset between 6 months and 10 years is possible [1, 8, 9]. In older dogs, cryptogenic epilepsy must be considered as a differential diagnosis for dogs showing epileptic seizures without any other abnormalities. Differentiation between idiopathic and cryptogenic epilepsy is difficult. A focal onset of seizures is considered a sign for the presence of an intracranial lesion, and thus for a cryptogenic epilepsy if no lesion is visible [7, 8, 37, 38]. Dogs in which a focal onset of seizures was described by the owner or was visible on the videotapes were not included in the study population. Additionally, only dogs in which the onset of the disease was between 1 and 3 years were included, making cryptogenic epilepsy unlikely.

In humans, an increase in the mean diffusivity in various brain regions, including white and gray matter, has been described with aging. These changes are thought to be due to Wallerian degeneration leading to gray matter loss and fiber alterations [39, 40]. Studies examining the effect of age on diffusion in the canine brain are lacking. Standard MRIs revealed changes in canine brain morphology similar to findings described for the human aging brain [41, 42]. A histopathological study examining the brains of young (1–5 years) and aged (8–18 years) dogs found changes identical to changes described in the aging human brain [43]. Therefore, it seems reasonable to assume similar changes in brain diffusion in dogs as described for humans.

However, exclusion of the two dogs exceeding the 6 year threshold (96 and 108 months, respectively) did not alter the results of our study, and therefore we decided to keep them in the study population.

As discussed previously for normal patients, the MRI system, vendor, magnetic field strength, coil and b-value can lead to variations in the ADC [32]. However, the settings for this study were identical to the described settings in healthy dogs. Thus, variations due to technical differences in the study design can be excluded as a cause of the differences in the ADC.

Overall a significant increase in diffusion as expressed by a higher ADC in the piriform lobe including the amygdala of diseased dogs (963.80 ± 174.69 μm^2^/s) compared with healthy dogs (915.44 ± 159.84 μm^2^/s) was found. Changes due to seizures are usually transient, but experimental data suggest that ADC changes may persist for days [30, 44, 45]. Changes in the piriform lobe and amygdala, which is located within the piriform lobe and is included in the ROI in this region, are only occasionally described in cases of epilepsy. Necrosis of the hippocampus and piriform lobe was described in a series of cats with epileptic seizures identified on post mortem histopathology. Unfortunately, no information about the time interval between the last seizure event and necropsy is provided in this study [46]. An experimental study in rats describes a decrease in the ADC 12 h after seizure induction [47]. After kainic acid-induced complex partial status epilepticus in dogs, an initial decrease in ADC, 3 and 6 h after seizure induction, with normalization after 48 h has been described in the hippocampus and amygdala. Unfortunately, it is unclear if the examinations in this study were performed during the ictus or after the seizures stopped [25]. Decreased diffusion during status epilepticus in various brain regions, including the piriform cortex and amygdala, has also been described by others in experimental studies using rats [48, 49]. Reduction in diffusion in human patients during the ictus and postictally is considered the result of a shift in water from the extracellular to the intracellular compartment, leading to cell swelling, thus reducing diffusion [30, 34, 35, 45, 50].

Interictal normalization of diffusion occurs in most cases [30, 34, 35, 45, 50]. However, some studies have described ADC changes in the hippocampus interictally with an increased ADC on the ictogenic side in human patients. The authors concluded that these changes were due to a loss of structural organization and an expansion of the extracellular space. We can only speculate about the reason for the increase in ADC in the piriform lobe including the amygdala; however, it seems reasonable to assume similar mechanisms as described for the hippocampus, with loss of structural organization and an expansion of the extracellular space.

We also encountered differences in diffusion in the hippocampus, with lower values in diseased dogs (1015.03 ± 231.79 μm^2^/s) compared with healthy dogs (1044.29 ± 165.21 μm^2^/s). However, these differences were not significant.

We can only speculate regarding possible causes of the increased ADC in the semioval center (diseased: 790.98 ± 229.63 μm^2^/s; healthy: 721.39 ± 126.28 μm^2^/s). An increase in the ADC in the subcortical white matter was described in 2/8 human patients, whereas the remaining 6/8 showed a decreased ADC. The reduced ADC was assumed to be the result of a vasogenic edema due to focally increased perfusion and vascular permeability. The authors of that study did not provide an explanation for the increased ADC they found in two patients [51]. Similar to the hippocampus, the semioval center is a small region and its borders could not always be clearly identified; thus, the inclusion of adjacent cerebral cortex might have occurred. The increased ADC values might therefore be due to an increased ADC of the cortex, rather than being related to an increased ADC of the semioval center. A relatively large standard deviation supports this theory. Although not significant, the ADC in the cerebral cortex in diseased dogs (875.65 ± 225.48 μm^2^/s) was higher than in healthy dogs (843.15 ± 98.24 μm^2^/s). The increased ADC of the cerebral cortex is possibly related to what is known as a functional deficit zone in human medicine. The functional deficit zone is defined as an area of the cortex that is functionally abnormal in the interictal period. Proposed mechanisms include a direct destructive effect of the epileptogenic zone on the deficit zone or a functionally mediated process such as abnormal neuronal transmission. However, the exact mechanism is not yet fully understood [52].

Otherwise, the recorded values were similar to those reported in healthy dogs [32]. Lower values were found for the thalamus (811.51 ± 175.96 μm^2^/s), reflecting its white and gray matter composition, and higher values were found for the caudate nucleus (871.47 ± 179.67 μm^2^/s), representing a gray matter structure.

Similar to healthy dogs, we found a significant difference between the right and left cerebral hemisphere in the ADC for the caudate nucleus and the piriform lobe including the amygdala. However, we did not encounter any tendency of a consistently higher diffusion value in one cerebral hemisphere in contrast to healthy dogs [32]. This finding might be due to differences in sex distribution in our study population of 12 males and 5 females in contrast to only 2 males and 8 females in the study of healthy dogs, leading to obfuscation of related differences.

The limitations of this study include the small study population, such that type 2 error cannot be excluded. Applying less strict inclusion criteria might have allowed for a larger study population. However, unacceptable bias would have occurred if the difference in case numbers between the study population (diseased dogs) and the control group of healthy dogs were larger. Another limitation is the heterogeneity of the study population with respect to age, breed and sex in comparison to the homogenous control group consisting only of Beagle dogs of similar age and body weight. In addition we cannot prove if changes are consequence or cause of epilepsy in this dogs.

## Conclusion

In summary, we found a significant increase in the ADC in the piriform lobe including the amygdala and the semioval center in dogs with IE compared with healthy dogs. One possible explanation for the increased ADC in the piriform lobe including the amygdala might be the loss of structural organization and an expansion of the extracellular space. The increased ADC in the semioval center might be related to the inclusion of part of the cerebral cortex in the ROI and a functional deficit zone. Despite the fact, that the encountered differences should be interpreted with caution interictal DWI seems to be a promising technique for the examination of canine epileptic patients lacking other gross neuromorphological abnormalities.

## Abbreviations

ADC: Apparent diffusion coefficient
ANOVA: Analysis of variance
DWI: Diffusion weighted MRI
IE: Idiopathic epilepsy
kg: Kilogram
Max: Maximum
Min: Minimum
MRI: Magnetic resonance imaging
ROI: Region of interest
S: Second
SD: Standard deviation
μm2: Micrometer squared

## Footnotes

1 Philips Intera Gyroscan, Philips Healthcare, Hamburg, Germany

2 SENSE-flex M coil, Philips Healthcare, Hamburg, Germany
